# High Peritoneal KT/V and Peritonitis Rates Are Associated with Peritoneal Calcification

**DOI:** 10.1371/journal.pone.0071636

**Published:** 2013-08-19

**Authors:** Jenq-Wen Huang, Yu-Chung Lien, Chung-Yi Yang, Kao-Lang Liu, Cheng-Chung Fang, Cho-Kai Wu, Jen-Kuang Lee, Hon-Yen Wu, Chih-Kang Chiang, Hui-Teng Cheng, Chung-Jen Yen, Kuan-Yu Hung

**Affiliations:** 1 Department of Internal Medicine, National Taiwan University College of Medicine and Hospital, Taipei, Taiwan; 2 Department of Medical Imaging, National Taiwan University College of Medicine and Hospital, Taipei, Taiwan; 3 Department of Integrated Diagnostics and Therapeutics, National Taiwan University College of Medicine and Hospital, Taipei, Taiwan; 4 Departments of Internal Medicine, Buddhist Tzu Chi General Hospital, Taipei Branch, New Taipei City, Taiwan; 5 Far Eastern Memorial Hospital, New Taipei City, Taiwan; 6 National Taiwan University Hospital, Hsin-Chu Branch, Hsin Chu City, Taiwan; 7 Cardiovascular Center & Department of Clinical Pathology, Far Eastern Memorial Hospital, New Taipei City, Taiwan; Cardiff University School of Medicine, United Kingdom

## Abstract

**Background:**

Peritoneal calcification (PC) is a specific finding in patients undergoing peritoneal dialysis (PD), but its prevalence, risk factors, and impacts in PD patients remain unclear. The present study investigated these issues and provided information useful for the management of PC.

**Methods:**

The study included 183 PD patients. The severity of PC was determined using abdominal computed tomography (CT), and we summed up all scores from slices obtained from the diaphragm to the pelvic floor normalized to body surface area. We analyzed the associations between PC and demographic and clinical characteristics, and between PC and levels of biomarkers, including C-reactive protein (CRP), osteoprotegrin and fetuin-A. The determinants of PC were examined using multiple regression analysis.

**Results:**

Patients were categorized into group 1 (without PC, n = 133) and group 2 (with PC, n = 50). Group 2 patients showed different degrees of PC with a mean of 160±769 mm^2^/m^2^. Group 1 patients had higher fetuin-A levels than group 2 patients (861±309 vs. 760±210 µg/mL; p = 0.021). The independent risk factors for the presence of PC included male gender, previous peritonitis, and PD adequacy (KT/V). Further analysis performed in group 2 patients showed that the dosage of vitamin D, serum levels of CRP, and dialysate calcium load were the independent determinants of PC. However, the presence of PC did not affect patients’ technique survival, peritonitis incidence, or mortality in the mean follow up period of 28±12 months.

**Conclusions:**

The presence and severity of PC were associated with inflammation, peritoneal KT/V, and mineral metabolism. The impact of PC on the outcomes of PD patients requires further study with a longer follow-up.

## Introduction

Peritoneal calcification (PC) is a rare finding in abdominal computed tomography (CT); however, peritoneal dialysis (PD) is associated with one-half of all benign PC cases [1]. Although calcium deposition is the major factor of PC, whether dysregulation of calcium-phosphate metabolism is a key factor in the pathogenesis of PD-related PC remains controversial [2], [3], [4], [5]. On the other hand, in hemodialysis (HD) patients, who commonly have mineral and bone disorders, PC is seldom detected. Therefore, uremia and secondary hyperparathyroidism cannot be considered as major factors involved in PC [6].

Factors specific to PD could be involved in the pathogenesis of PC. However, there is a lack of a large study aimed at defining the risk factors of PC. Several case reports proposed possible links between PC and PD, including calcium from dialysate [7], repeated peritonitis [2], [8], calciphylaxis [9], and hypertonic dialysate [10]. These are all local factors in the peritoneal cavity. A recent study showed that matrix Gla protein, a calcification inhibitor synthesized by the mesothelium, was up-regulated by high glucose levels [11]. This phenomenon may occur in response to exposure to unphysiologic dialysate rather than as a protective effect of high glucose per se because it is not compatible with clinical observations [10]. Fetuin-A and osteoprotegerin (OPG) are two biomarkers related to the calcification in ESRD patients. Lower fetuin-A levels are associated with a lower arteriovenous fistula patency rates [12] in HD patients. In PD patients, lower fetuin-A levels are also linked to mortality and CVD events [13]. OPG suppression is associated with arterial and valvular calcification, indicating an anti-calcification activity of OPG [14]. However, OPG is elevated and associated with more severe calcification in ESRD patients [15], [16]. Therefore, the role of OPG in PC among PD patients remains unclear.

The casual relationship between these factors and PC cannot be inferred simply from the case reports. For example, whether hypertonic dialysate applied in high transporter patients leads to PC or PC results in a high transporter with subsequent use of hypertonic dialysate remains unclear.

The impact of PC on PD has not been investigated thoroughly, although PC has been hypothesized to advance to encapsulating peritoneal sclerosis (EPS), the most catastrophic complication of PD. The “two hits theory” of EPS comprising of the first hit of bioincompatible dialysate and the second hit of peritonitis [17] can be applied to PC. Some EPS cases present with PC [18], [19], but PC is not a mandatory criterion for EPS. No large studies have investigated the prevalence and severity of PC among PD patients.

This study was designed to investigate these issues and provide a method to quantify the severity of PC using CT. The aims of the present study were to determine the real prevalence and the associated factors of PC, and to clarify whether PC has an impact on the outcomes of PD patients.

## Materials and Methods

### Study Design

Patients older than 20 years of age who had undergone maintenance PD for more than 3 months at National Taiwan University Hospital were invited to join the present study that began in 2009. Pregnant woman and patients who had undergone CT within the previous 6 months were excluded. After providing informed consent to participate, each patient underwent non-contrast abdominal CT. The blood and PD effluent samples were obtained during the routine peritoneal equilibration test (PET) nearest the date of the CT scan for biomarker evaluation. The blood samples were centrifuged immediately at 3000 rpm at 4°C, and the plasma supernatants as well as the PD effluent were frozen at −80°C until measurement.

### Ethical Considerations

This study was approved by ethics committee of National Taiwan University hospital (approval number, NTUH-REC No. 200808062R). All patients provided written informed consent to participate before being enrolled in the study.

### Assessment of Peritoneal Calcification

**Figure 1:**
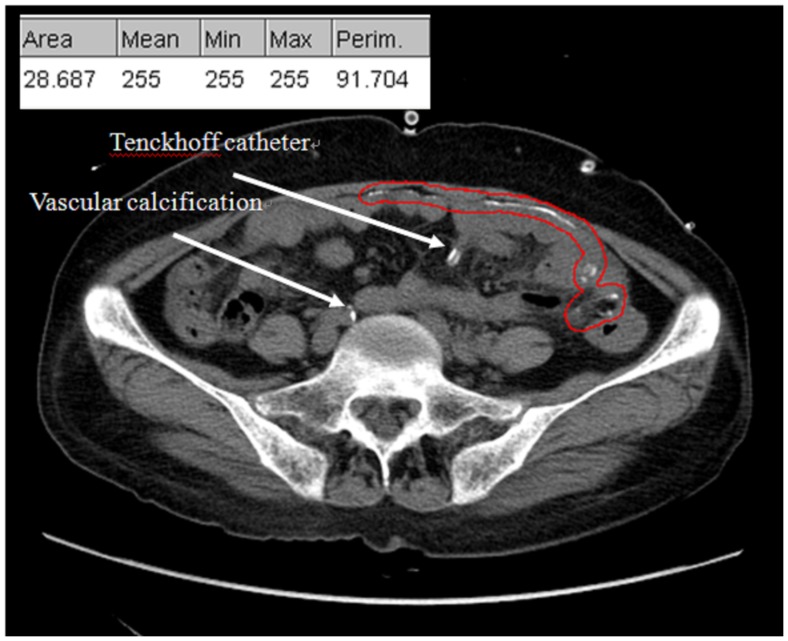
The method of measuring peritoneal calcification (PC). PC located in the abdominal wall and bowel wall was circled as the region of interest. The software ImageJ quantified the calcification areas of more than 150 Hounsfield units (table in the figure). The Tenckhoff catheter and vascular calcification were excluded from measurement.

Imaging was performed using a 64-MDCT scanner (LightSpeed VCT; GE Healthcare, Milwaukee, Wis). Image analysis software (ImageJ, version 1.45; National Institutes of Health, Bethesda, Md) was used with an attenuation range of more than 150 Hounsfield units to quantify the calcification area within the region of interest (Fig. 1). PC was detected in the parietal peritoneum (abdominal wall) or parietal peritoneum (bowel wall or liver surface), as shown in the CT scan. Vascular calcification, Tenckhoff catheter, bony structures, and the content of the lumen of the gastrointestinal tract were all carefully excluded. The measurement was performed on each slice of the CT scan to image the entire PC area in the abdominal cavity (from diaphragm to rectovesical pouch). The calcification areas of each slice were added and normalized to the body surface area (BSA) to represent the severity of PC in each patient. The images were reviewed independently by 2 radiologists (CY Yang and KL Liu) who were blinded to the clinical characteristics of the PD patients.

### Clinical Characteristics and Follow-up

The clinical and dialysis-related parameters of the participants, including PET results, dialysis Kt/V, residual renal function, and normalized protein catabolic rate (nPCR), were all recorded. The dialysate used in 2009 was also recorded to calculate the dialysate glucose and calcium load, and quantify the annual exposure as previously reported [20], [21], [22]. For patients who dropped out in 2009, the annual amount was corrected using the PD duration during the year of the study. Routine biochemical parameters, including calcium, phosphate, alkaline phosphatase (ALP), parathyroid hormone (PTH), blood urea nitrogen (BUN), creatinine, albumin, hemoglobin, and C-reactive protein (CRP) levels were also recorded. Medication histories were obtained, including the monthly dose of calcium-based phosphate binder and vitamin D doses received 3 months before the CT scan. Other aspects of the medical histories, such as parathyroidectomy and peritonitis episodes occurring before the CT scan were also recorded. Patients were prospectively evaluated after the CT scan for the occurrence of the following events: hospitalization, peritonitis, transplant, technique failure, and mortality. The follow up ended in February 2012.

### Biomarker Measurement

The fetuin-A (R&D Systems, Minneapolis, USA) and OPG (RayBiotech, Norcross, GA) levels in the plasma samples were measured using commercially available enzyme-linked immunosorbent assay (ELISA) kits according to the manufacturers’ instructions.

### Statistical Analysis

All continuous variables are reported as mean ± standard deviation (SD) (with 95% confidence intervals where appropriate), and all categorical variables are reported as frequencies or percentages. The Student’s *t*-test was used to analyze between-group differences as appropriate. Differences in frequencies were tested using chi-square analysis. Relationships between variables were tested using Pearson’s correlation. Poisson analysis was used to compare the incidences of peritonitis and morbidity. The independent determinants of given variables were analyzed using multiple regression analysis. The adjusted variables were stated in each analysis. Kaplan-Meier survival analysis was used to compare outcomes between groups. P values <0.05 were considered significant. The statistical analyses were performed using SPSS 13.0 for Windows (SPSS Inc., IL, USA).

## Results

### Clinical and PD Associated Characteristics

A total of 183 PD patients (84 men and 98 women; mean age, 53±13 years; mean PD vintage, 41±38 months) were enrolled in this study. Fifty (27%) patients had CT-detectable PC. We categorized all the PD patients into 2 groups for further comparisons. Group 1 patients had no PC (n = 133), and group 2 included patients with PC (n = 50). Group 2 patients had significantly lower BUN, hemoglobin, fetuin-A levels than group 1 patients (Table 1). In addition, group 2 patients had lower albumin levels and lower dosages of calcium-based phosphate binder than group 1 patients. Moreover, group 2 had fewer female patients than group 1.

**Table 1:** Comparisons of the clinical characteristics of peritoneal dialysis (PD) patients without (group 1) and with (group 2) peritoneal calcification (PC).

	Group 1(no PC,n = 133)	Group 2(PC, n = 50)	P
Women	76 (57%)	22 (44%)	0.078
Parathyroidectomy	18 (14%)	5 (10%)	0.404
Age (years)	53±12	53±15	0.997
PD vintage (months)	40±34	44±48	0.503
Albumin (g/dL)	4.0±0.4	3.9±0.4	0.073
Hemoglobin (g/dL)	10.3±1.3	9.7±1.3	0.009
Urea nitrogen (mg/dL)	60±15	54±14	0.012
Creatinine (mg/dL)	11.2±2.7	11.4±2.9	0.707
Calcium (mg/dL)	9.6±1.0	9.6±0.8	0.870
Phosphate (mg/dL)	5.4±1.3	5.4±1.2	0.923
Alkaline Phosphatase (IU/L)	216±120	247±145	0.138
PTH (pg/ml)	353±282	419±440	0.236
Fetuin A (ug/ml)	861±309	760±210	0.021
Osteoprotegrin (pg/ml)	961±518	1062±1209	0.466
CRP (mg/dL)	1.17±2.51	0.96±1.14	0.561
Ca phosphate binder (g/month)	51±35	41±28	0.063
Calcium phosphate binder(g/month)	51±35	41±28	0.063
Vit. D dose (µg/month)	22±32	18±29	0.455
Comorbidity			
Cardiovascular disease	30 (23%)	7 (14%)	0.223
Diabetes	34 (26%)	11 (22%)	0.385
Hypertension	116 (87%)	37 (74%)	0.149
Cause of ESRD			0.302
Glomerulonephritis	66 (50%)	28 (56%)	
Hypertension	11 (8%)	0	
Diabetes	28 (21%)	11 (22%)	
Cystic kidney disease	3 (2%)	2 (4%)	
Others	25 (19%)	9 (18%)	

CRP, C-reactive protein; PTH, parathyroid hormone; PD, peritoneal dialysis; ESRD, end-stage renal disease.

Group 2 patients had a mean PC of 160 mm^2^/m^2^ with wide variation. With regard to PD associated parameters, group 2 patients used a higher annual amount of glucose dialysate and total dialysate, and had higher peritoneal KT/V than group 1 patients (Table 2). The dialysate glucose and calcium load was similar between the 2 groups. Prior to the CT scan, the average peritonitis incidence was significantly higher in group 2 patients than in group 1 patients (1.93±3.5 vs. 0.88±1.82 episodes/100 patient-months; p = 0.009).

**Table 2:** Comparisons of the PD associated characteristics of the 2 groups without (group 1) and with (group 2) peritoneal calcification (PC).

	Group 1(no PC, n = 133)			Group 2(PC, n = 50)			P
nPCR (g/Kg/day)	0.96±0.19			0.94±0.21			0.499
4 h D/P creatinine	0.66±0.09			0.68±0.12			0.244
D4/D0 Glucose	0.39±0.07			0.37±0.08			0.156
Renal KtV	0.22±0.33			0.13±0.19			0.098
Peritoneal KtV	1.83±0.35			1.98±0.40			0.015
Glucose dialysate (L/year)	2878±859			3307±997			0.005
Total dialysate (L/year)	3240±833			3578±919			0.019
Ca exposure	157±49			180±60			0.008
(glucose dialysate, g/year)							
Total Ca exposure	182±48			199±57			0.050
(total dialysate, g/year)							
Calcium load	5.7±0.6			5.6±0.6			0.459
(glucose dialysate, mg/dL)							
Calcium load	5.5±0.6			5.5±0.6			0.914
(total dialysate, mg/dL)							
Glucose exposure (Kg/year)	55±19			62±24			0.056
Glucose load	1.93±0.34			1.92±0.32			0.964
(glucose dialysate, g/dL)							
Glucose load	1.69±0.29			1.76±0.34			0.148
(total dialysate, g/dL)							
Previous peritonitis rate^a^	0.88±1.82			1.93±3.50			0.009
(/100 patient months)							
PC/BSA (mm^2^/m^2^)				160±769			

nPCR: normalized protein catabolic rate.

a Average of peritonitis incidence of each patient.

### Independent Predictors of PC

To predict the occurrence of PC, we further analyzed the independent determinants using multiple logistic regression, and found that peritoneal KT/V and prior history of peritonitis increased the risk of PC. In addition, female patients had a lower odds ratio of occurrence of PC after adjusting for PD vintage than male patients (Table 3).

**Table 3:** Independent determinants for presenting PC or not using multiple logistic regression analysis with adjustment for PD vintage.

	OR	95.0% C.I.	P
Peritoneal KtV	5.23	1.50∼18.31	0.010
Women	0.38	0.18∼0.80	0.011
Prior peritonitis(/100 patient months)	1.17	1.03∼1.34	0.019
PD vintage	1.00	0.99∼1.01	0.656

### Factors Affecting the Severity of PC

The incidence of PC in group 2 patients was variable, and we used log transformation of PC for further analysis. The relationship between other parameters and PC was further analyzed using Pearson’s correlation. BUN and dialysate calcium load negatively correlated with PC, and oral vitamin D dose, dialysate glucose load, and serum calcium and CRP levels positively correlated with PC (Table 4). PD parameters, inflammation, and medication correlated with the severity of PC. The independent determinants for PC were further explored using multiple linear regression. Oral vitamin D dose and CRP level were independent positive determinants for PC (Table 5). In contrast, dialysate calcium load was a negative predictor of PC. Although the number of variables was limited by the small number of cases included, the predictability of these 3 determinants was 0.422.

**Table 4:** Correlations between the severity of peritoneal calcification and clinical or PD associated characteristics in patients with PC (n = 50).

	Ln(PC/BSA)	P
Sex	0.02	0.879
DM	−0.07	0.611
Parathyroidectomy	−0.26	0.073
Age	0.23	0.103
PD vintage	0.11	0.435
Albumin	−0.08	0.580
Hemoglobin	−0.13	0.377
Urea nitrogen	−0.29	0.041
Creatinine	−0.14	0.346
Calcium	0.28	0.053
Phosphate	−0.06	0.703
Alkaline Phosphatase	−0.05	0.752
Parathyroid hormone	0.20	0.160
Fetuin A	0.25	0.112
Oseoprotegrin	−0.11	0.480
LnCRP	0.35	0.013
Calcium phosphate binder	−0.09	0.539
Vit. D dose	0.48	0.000
4 h D/P creatinine	0.09	0.529
Renal KtV	−0.24	0.094
Peritoneal KtV	0.02	0.886
Annual glucose dialysate	0.14	0.327
Annual total dialysate	0.10	0.513
Annual Ca exposure (glucose dialysate)	0.03	0.824
Annual Ca exposure (total dialysate)	−0.03	0.854
Calcium load (glucose dialysate)	−0.37	0.008
Calcium load (total dialysate)	−0.35	0.013
Annual glucose exposure	0.25	0.081
Glucose load (total dialysate)	0.35	0.012
Glucose load (glucose dialysate)	0.29	0.041
Previous peritonitis rate	−0.18	0.204

The severity of PC was summed PC area on the CT scan normalized to body surface area. Therefore, the value was log transformed.

**Table 5:** Independent determinants of the severity of PC, in patients with detectable PC on CT scan, using multiple linear regression analysis.

	B±SE	95% CI	P
Constant	5.56±2.24	1.06∼10.06	0.013
Vit. D dose (per 10 µg/month)	0.31±0.08	0.15∼0.47	<0.001
Calcium load(total dialysate, per mg/L)	−0.87±0.39	−1.65∼−0.01	0.032
LnCRP	0.40±0.14	0.12∼0.67	0.005
R^2^	0.422		

The severity of PC was summed PC area on the CT scan normalized to body surface area. Therefore, the value was log transformed.

### Influence of PC

As PC involves changes in peritoneal anatomy, the impact of PC on PD was further investigated. Group 2 patients had a higher incidence of peritonitis prior to the CT scan than group 1 patients (1.62 vs. 1.05/100 patient-month; p<0.001 using Poisson analysis, Table 6). After CT scan, the 2 groups had a similar mean follow-up duration (29±10 vs. 28±12 months) and showed similar rates of peritonitis and hospitalization during this period. In addition, the outcomes were not significantly different between the 2 groups. Twelve patients switched to HD in group 1 (10 for refractory peritonitis, 1 for ultrafiltration failure and 1 for hernia) and 9 patients switch to HD in group 2 (6 for refractory peritonitis, 1 for omentum wrapping, and 2 for hernia). In the follow-up period, 3 patients in group 1 developed EPS after episodes of refractory peritonitis. The CT scan revealed loculated ascites and variable diameters of bowel loops with thickened walls. One patient died of severe infection, and the other 2 recovered with treatment of tamoxifen and steroids after catheter removal. These following CT scans taken during the episode of EPS still revealed no PC.

**Table 6:** Comparison of various outcomes and morbidity targets between the 2 groups.

	Group 1(no PC, n = 133)	Group 2(PC, n = 50)	P
Before CT scan			
Total PD duration (months)	5342	2220	
Peritonitis episodes	56	36	
Peritonitis rate(/100 patient-month)	1.05	1.62	<0.001
After CT scan			
Total follow up duration(months)	3901	1421	
Peritonitis episode	56	17	
Peritonitis rate(/100 patient-month)	1.44	1.20	0.27
Hospitalization rate(/100 patient-month)	4.61	5.28	0.89
Outcomes (%)			0.41
PD	92 (68%)	34 (68%)	
Hemodialysis	12 (9%)	9 (18%)	
Transplant	14 (11%)	4 (8%)	
Death	14 (11%)	3 (6%)	
Recovery	1 (1%)		

**Figure 2:**
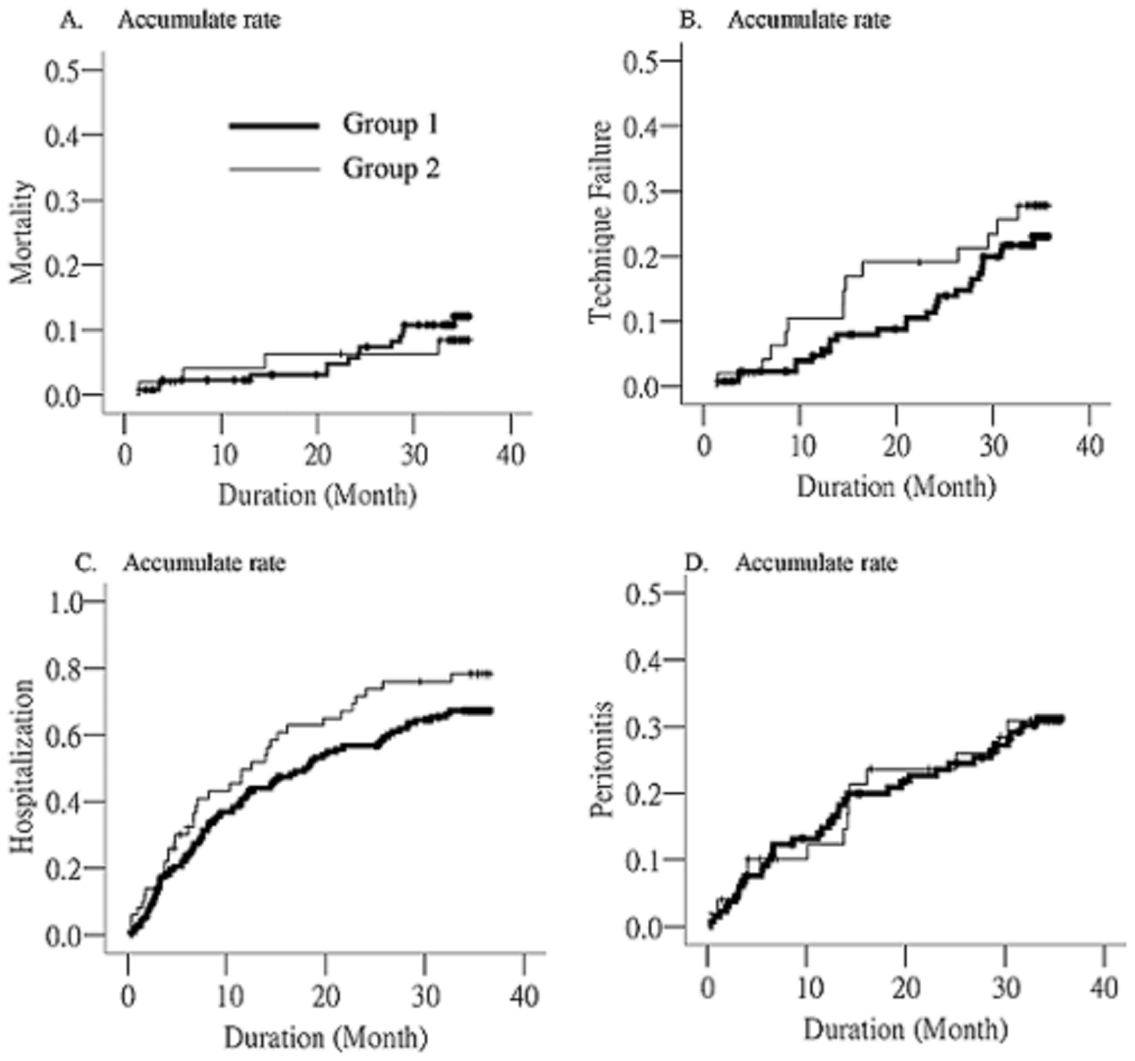
Kaplan-Meier survival analysis was used to compare outcomes between patients without (group 1; n = 133) and with (group 2; n = 50) peritoneal calcification. There was no significant difference between the 2 groups in event free survival of mortality (A), technique failure (B), hospitalization (C), and peritonitis (D).

In Kaplan-Meier survival analysis, the interval to mortality, technique failure, hospitalization, and peritonitis were not different between the 2 groups (Fig. 2).

## Discussion

The present study is the first large-scale study to investigate the severity of PC in PD patients. We found that risk factors for PC were male gender, higher peritoneal KT/V, and individual peritonitis incidence rates. The severity of PC was associated with oral vitamin D dose, dialysate calcium load, and CRP level. However, PC was not associated with short-term patient outcomes with respect to mortality, technique failure, hospitalization, or subsequent peritonitis.

In a prevalent PD population with a mean dialysis vintage of 41 months, 27% of patients were diagnosed with PC. PC patients had a higher individual frequency of peritonitis than non-PC patients. Peritonitis episodes induce vigorous inflammatory reactions in the peritoneum. In vascular smooth muscles, interleukin 6 and tumor necrosis factor alpha can act as pro-calcific cytokines to induce vascular calcification in a paracrine fashion [23]. The same mechanism could occur in peritonitis, resulting in the production of more inflammatory cytokines leading to calcification in the nearby peritoneum. The inflammatory hypothesis is supported by our findings that show a correlation between high CRP levels and increased severity of PC (Table 5).

The second factor associated with PC was the peritoneal KT/V. Group 2 patients used more dialysate and had a higher annual exposure to calcium and glucose than group 1 patients (Table 2).These parameters can be attributed to the high peritoneal KT/V. On the other hand, from a clinical aspect, patients with a low renal KT/V required a higher peritoneal KT/V to achieve PD adequacy. These results underscored the importance of residual renal function in PD patients.

The content of dialysate was also important. A hypertonic glucose concentration will induce fibrosis in the peritoneum [24], [25] and can result in poor survival as determined in our previous studies [20], [21], [22]. A hypertonic dialysate had been implicated as a risk factor for PC in a previous case report [10]. In the present study, glucose load significantly correlated with the severity of PC, implying that a hypertonic dialysate was at least an aggravating factor for PC (Table 4).

The third factor associated with PC was mineral metabolism disorders, which are a common problem in dialysis patients. Vascular calcification has been added in the new classification of this disorder [26]. PC occurring as vascular calcification is also an ectopic calcification. In our study, mineral parameters were not different in both groups except PC patients had lower fetuin A levels (Table 1). Fetuin-A is an important inhibitor of extraosseous calcification. Low fetuin-levels are associated with inflammation, vascular calcification, and a lower arteriovenous fistula patency rates in HD patients [12], [27] and might be also related to PC in PD patients from our observation.

Vitamin D dose and dialysate calcium load were independent determinants of the severity of PC, showing opposite effects (Table 5). High doses of vitamin D may result in greater suppression of PTH levels. However, a bimodal effect of vitamin D has been postulated with regard to the regulation of ectopic calcification, i.e., physiological doses of vitamin D ameliorated calcification whereas high doses aggravated calcification [28]. A similar result was obtained in our study, where high vitamin D doses were positively associated with the severity of PC. In contrast, dialysate calcium load was a negative predictor, whereas serum calcium levels positively correlated with the severity of PC (Table 4). Further analysis revealed that serum calcium levels were strongly negatively correlated with dialysate calcium load (r = −0.42; p<0.001) in these PC patients. These results suggested that the source of calcium for PC was the blood instead of the dialysate.

**Figure 3:**
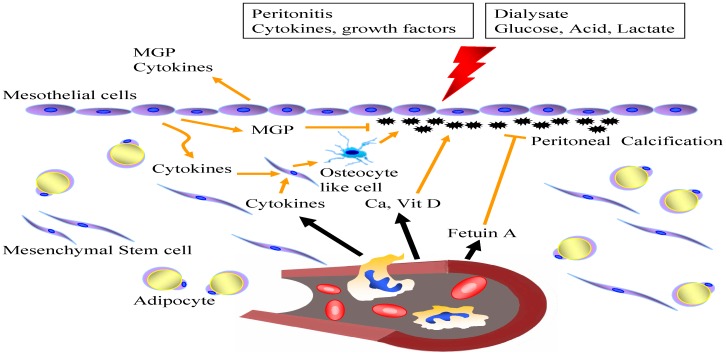
Proposed pathogenesis of peritoneal calcification. Unphysiologic dialysate and peritonitis episodes stimulate mesothelial cells which secret calcification inhibitors (matrix Gla protein, MGP) and other cytokines. Some other calcification inhibitor (fetuin A) and cytokines are also across the capillary wall from the circulation. These cytokines might transform the mesenchymal cells into osteocyte-like cells to induce following calcification. In addition, blood provide calcium and vitamin D to facilitate calcification.

Base on the results of this study, a hypothesis of pathogenesis about PC is proposed (Fig. 3). Dialysate and peritonitis stimulate mesothelial cells which secret calcification inhibitors (ex. matrix Gla protein) and other cytokines. From the pathogenesis of vascular wall calcification, osteocyte-like cells are mandatory in the process of calcification [29]. These cytokines might induce the mesenchymal cells to transform into osteocyte-like cells to induce calcification. Calcium is derived from blood in this process instead of dialysate.

Some authors suspect that PC is associated with EPS [18], [19], but the definite impact of PC on EPS has never been evaluated. Three patients in group 1 without PC in this study developed EPS caused by refractory peritonitis in the follow-up period. The CT scans taken during the EPS episodes, still did not detect PC. The impact of PC on EPS cannot be obtained from present study since the EPS case number was only 3. In addition, although PC patients have a historical higher incidence of peritonitis, PC did not make patients prone to peritonitis in the follow-up period. We were unable to determine the effect of PC on the outcomes of PD patients with regard to morbidity, technique failure, and mortality. As the severity of PC varied widely, previous study only provide a semi-quantitative measurement of PC [30]. We propose a useful method to quantify the PC area, but CT scan is not a common study equipment. Since the change of peritoneum should develop after a long period PD, the impact of PC could not be explored in the limited followed-up period of 28 months in this study.

In conclusion, PC was detected in 27% of prevalent PD patients. High frequency of peritonitis, peritoneal KT/V, and male gender were the risk factors for PC. Vitamin D dose, inflammation, and dialysate calcium load were the predictors of the severity of PC. Both mineral metabolism and inflammation were associated with the pathogenesis of PC. Although PC involves an anatomical change, the PD technique survival was not affected by PC. The present study provided preliminary results; however, the elucidation of the pathogenesis and impact of PC requires further investigation.
